# Removing One Barrier to Protecting Sex Partners in HIV Remission Studies With a Treatment Interruption

**DOI:** 10.1093/infdis/jiz162

**Published:** 2019-07-02

**Authors:** Nir Eyal

**Affiliations:** Center for Population-Level Bioethics, Rutgers University, New Brunswick, New Jersey

**Keywords:** HIV, research ethics, analytic treatment interruption, HIV cure-related studies, clinical trials, human subject research

## Abstract

Only a few of the recommendations made in this supplement are implemented in ongoing HIV remission studies that involve an analytical treatment interruption. The absence of these recommendations in protocols puts sexual partners of study participants at serious risk. This paper addresses one possible barrier to implementation: a certain misunderstanding among sponsors and research entities.

This supplement recommends some measures to help protect participants’ sexual partners from human immunodeficiency virus (HIV) infection in HIV remission studies with an analytical treatment interruption (ATI), and to keep any remaining risks ethically defensible.

It is noteworthy that only some of the recommended measures are part of either current or completed protocols [1]. This contrasts with how researchers and sponsors tend to address participant infection in, for example, HIV prevention trials—where participants usually receive HIV counseling and a “standard of prevention” package of this or that form [2] throughout the trial. There are probably many reasons why. Perhaps researchers, sponsors, or ethics reviewers have different views than ours on what protections would be appropriate. Perhaps they have not yet thought through these matters (research ethics is largely dedicated to protecting study participants, not nonparticipants). Perhaps they know that legally they can get away with neglecting their protection (although some of the measures currently not implemented would be cheap). Empirical research could be done to help explain this relative paucity of protections. But in conversations with researchers, I received anecdotal indications that there may be a further barrier. Research teams and especially their drug company sponsors are loath to be seen to take ownership over potential HIV infections in any way. They therefore avoid measures to prevent infections, lest the inadvertent occurrence of such infections (which cannot be stemmed completely) create legal, regulatory, or public opinion trouble for sponsoring companies and research institutes.

I do not know how central this alleged barrier is in practice, but what follows explains why it would be based largely on sheer misunderstanding. Clearing such potential misunderstanding matters, because it may help protect sexual partners, and keep any remaining risk to them defensible.

Four clarifications may help ease worries about legal, regulatory, and public opinion trouble following protective measures. First, 45 CFR 46, which formally governs US federally funded research and creates informal expectations across clinical research in the US and beyond, demands no compensation for injuries to study participants [3, 4]. This may be ethically regrettable [4, 5], but it is the case, especially in the jurisdiction in which most controlled HIV remission studies with an ATI take place.

Second, nonparticipants are not legally entitled to any of the protections included in 45 CFR 46, precisely because they are not study participants [3]. Sexual partners, in particular, lack legal rights to injury compensation, and to other human subjects protections and privileges, for example special privacy protections, consent rights, and so forth (45 CFR 46 affords fetuses some minimal protections) [6]. (An alternative account of the relative paucity of protections for participants’ sexual partners might have been that sponsors know that legally they can get away with failure to protect partners; yet even low-cost protections like partner counseling are currently rarely provided. For sponsors, providing such protections would have trifling costs compared to the financial stakes and improved relations with advocates for people living with HIV. The possibility of a misunderstanding discussed herein occurred to me following conversations with researchers who had discussed sexual partner protection with sponsors. On balance, that possibility strikes me as somewhat more plausible.)

A third clarification addresses any fear that regulators like the US Food and Drug Administration (FDA) would take injuries to third parties to count against the drugs being tested in the curative trial, and block their approval or their off-label testing. Surely the FDA would have the medical expertise to judge that, unlike potential injuries to study participants, who are sometimes on experimental drugs, any onward transmission of HIV during antiretroviral therapy (ART) interruption is far likelier to have resulted from infectiousness created by the interruption of ART than from interaction with the drugs being tested.

A final point is relevant in case the worry stems from speculation about public anger at the researchers or the sponsors in the event of injury. A no less plausible speculation is that public anger at researchers and sponsors who went out of their way to prevent onward transmission would be less. To avoid important protections on the questionable speculation that, in the event of an infection, having attempted to protect nonparticipants would somehow make one’s company look bad is both cynical and imprudent.

In short, sheer misunderstanding may currently be the barrier to implementing some of the measures recommended in this supplement. We hope that this commentary helps clear this potential misunderstanding and open up one bottleneck to ethical treatment of nonparticipants at risk from important HIV remission studies.

## Notes

Presented in part: Medical Study Risks to Nonparticipants: Ethical Considerations Workshop, Harvard T.H. Chan School of Public Health, Boston, MA, 11–12 June 2017.

## Notes

***Acknowledgment.*** The author gratefully acknowledges helpful comments and editorial assistance from Lisa Holtzman and Monica Magalhaes.

***Financial support.*** This supplement is sponsored by NIH: National Institute Allergy and Infectious Diseases [grant number 1 R01 AI114617-01A1 (HIV cure studies: risk, risk perception, and ethics)].

***Potential conflicts of interest.*** The author certifies no potential conflicts of interest. The author has submitted the ICMJE Form for Disclosure of Potential Conflicts of Interest. Conflicts that the editors consider relevant to the content of the manuscript have been disclosed.
